# Multiphysics-Coupled Load-Bearing Capacity of Piezoelectric Stacks in Low-Temperature Environments

**DOI:** 10.3390/s25123642

**Published:** 2025-06-10

**Authors:** Yang Li, Yongping Zheng, Leipeng Song, Zhefan Yao, Hui Zhang, Yonglin Wang, Zhengshun Fei, Xiaozhou Xu, Xinjian Xiang

**Affiliations:** 1 School of Automation and Electrical Engineering, Zhejiang University of Science and Technology, Hangzhou 310023, China; 120048@zust.edu.cn (Y.L.); zyp10024@163.com (Y.Z.); 124018@zust.edu.cn (L.S.); zsfei@zust.edu.cn (Z.F.); helenxu@zust.edu.cn (X.X.); 2 Laisiao Integrated Home Furnishing Co., Ltd., Jiaxing 314011, China; 18867682600@163.com (Z.Y.); hui2125@163.com (H.Z.); wyljxzj@163.com (Y.W.)

**Keywords:** piezoelectric stack actuators, thermo-electro-mechanical coupling, load-bearing capacity

## Abstract

Under low-temperature conditions, the load-bearing capacity of piezoelectric stacks arises from coupled thermo-electro-mechanical interactions, with temperature fluctuations, compressive prestress, and excitation voltage critically modulating performance. This study introduces an integrated measurement platform to systematically quantify these interdependencies, employing a cantilever-based sensing mechanism where bending strain serves as a direct metric of load-bearing capacity. A particle swarm-optimized theoretical framework guides the spatial configuration of actuators and sensors, maximizing strain signal fidelity while suppressing noise interference. Experimental characterization reveals three key findings: 1. Voltage-dependent linear enhancement of load-bearing capacity across all operational regimes, unaffected by thermal or mechanical variations; 2. Prestress-induced amplification (79–90% increase from 0 to 6 MPa) and thermally driven attenuation (15–30% reduction from 20 to −70 °C) of static performance; 3. Frequency-dependent degradation (1–6 Hz) in dynamic load-bearing capacity. The methodology establishes a robust foundation for designing multiphysics-compatible instrumentation systems, enabling precise evaluation of smart material behavior under extreme coupled-field conditions.

## 1. Introduction

Piezoelectric stack actuators (PSAs) have become pivotal elements in contemporary intelligent control architectures, distinguished by their exceptional energy transduction efficiency, broad operational bandwidth, and rapid transient responsiveness, positioning them as indispensable components among smart material-based actuation technologies [1,2,3,4]. These actuators are increasingly deployed in robotics, unmanned systems, and industrial automation, where precision and adaptability are paramount [5,6]. For instance, in robotic joint control, PSAs enable micro-level adjustments for delicate tasks such as surgical robotics [7], while in unmanned aerial vehicles (UAVs), they drive adaptive wing morphing mechanisms to enhance aerodynamic performance under dynamic environmental conditions [8]. Furthermore, in industrial automation, PSAs are integral to high-precision assembly lines, where their ability to generate large forces with nanometer-scale resolution ensures robust control of manufacturing processes [9]. The growing demand for PSAs in extreme environments, particularly low-temperature applications, highlights their versatility in intelligent control scenarios. Examples include active vibration suppression systems in cryogenic wind tunnels (e.g., liquid nitrogen-cooled aerospace testing facilities [10,11,12]) and force feedback mechanisms in superconducting cavities operating near absolute zero [13]. In low-temperature environments, reduced piezoelectric coefficients and complex domain dynamics degrade actuator efficiency [14].

The inherent limited tensile strength of piezoelectric ceramics necessitates the application of compressive prestress during PSA operation to mitigate tensile failure risks. Notably, such preloading conditions induce complex interdependencies between mechanical constraints and electromechanical response, as the imposed prestress alters domain wall dynamics and crystal phase transitions within the piezoceramic microstructure [15,16]. Concurrently, the operational efficacy of PSAs is intrinsically governed by the applied voltage levels, in accordance with fundamental piezoelectric principles. In low-temperature environments, these actuators are subjected to coupled thermo-electro-mechanical (T-E-M) stimuli, where temperature, prestress magnitudes, and excitation voltages collectively modulate displacement characteristics. This multi-physics interplay not only dictates transient load-bearing capacity but also determines the long-term stability of PSA-integrated systems, such as cryogenic positioning mechanisms or adaptive robotic joints. Consequently, elucidating the synergistic mechanisms underlying T-E-M field interactions holds critical implications for optimizing PSA performance metrics-including stroke efficiency, hysteresis reduction, and fatigue resistance-across applications ranging from aerospace actuation to precision manufacturing.

Existing research efforts have predominantly explored the multi-physical coupling effects on PSA behavior under unconstrained configurations, with a focus on free-state displacement outputs [8,17,18] or intrinsic material parameters like piezoelectric coefficients and electromechanical coupling factors [19,20]. Such measurements, under standardized conditions, can be facilitated by instrumentation including specialized commercial platforms like the Aixacct AIXCMA, designed for quantifying unconstrained responses and relevant piezoelectric parameters. Notably, experimental investigations under extreme thermal conditions remain limited, as the technical challenges associated with low-temperature instrumentation design often restrict such studies to elevated temperature regimes. For example, Mitrovic et al. [21] systematically characterized strain responses, dielectric properties, and stiffness variations in PSAs subjected to isolated or combined electrical/mechanical loads, yet their work omitted subzero temperature analysis. Similarly, Li et al. [22] evaluated quasi-static T-E-M interactions within a thermal range of −30 °C to 125 °C, demonstrating stroke dependencies on electric field strength (0.3–1.8 kV/mm) and prestress (4.6 MPa), but this scope excluded cryogenic operating conditions critical for aerospace or superconducting applications. However, the low-temperature performance of PSAs demands rigorous investigation [17,23,24], as subzero temperatures alter domain dynamics and strain transfer-key determinants of their reliability in aerospace and robotic applications.

However, a critical challenge remains in detecting and understanding the load-bearing capacity of PSAs under coupled T-E-M fields. Crucially, prior investigations rarely address this key performance metric-such as force transmission capabilities under structural constraints or system-level performance degradation in mechanically coupled environments-despite its centrality to real-world implementations like adaptive robotics, aerospace actuation, and cryogenic systems. This knowledge gap is compounded by the lack of dedicated instrumentation for evaluating PSA performance, particularly load-bearing capacity, under concurrent low-temperature, electrical, and mechanical stimuli. These limitations hinder the optimization of PSA-driven systems requiring synchronized precision and robustness in multi-field environments, ultimately impacting the reliability and viability of intelligent actuation systems where complex T-E-M interdependencies dictate performance.

To bridge this gap, this study systematically investigates the coupled T-E-M interactions governing the load-bearing capacity of PSAs. To decouple multi-physics dependencies, an innovative instrumentation system was developed, employing a cantilever beam as the sensing platform to quantify the PSA’s load-bearing capacity through strain-induced bending responses, while applying mechanical load via a gravitationally amplified lever system. This system enables independent modulation of temperature (−70 to 20 °C), prestress (0 to 6 MPa), and excitation voltage (DC/AC 0 to 120 V), thereby providing quantitative insights into the load-bearing capacity of PSAs under extreme multi-field coupling and effectively addressing this critical knowledge gap. Furthermore, the integration of a particle swarm-optimized theoretical model, strain transfer analysis, and low-temperature mechanical testing protocols establishes a comprehensive framework that bridges existing qualitative observations with quantitative metrics-a critical advancement for optimizing PSA reliability in load-adaptive systems such as aerospace actuators and robotic manipulators.

## 2. Methodology for Assessing PSA Load-Bearing Capacity

### 2.1. Analytical Framework for PSA-Driven Cantilever Systems

**Figure 1:**
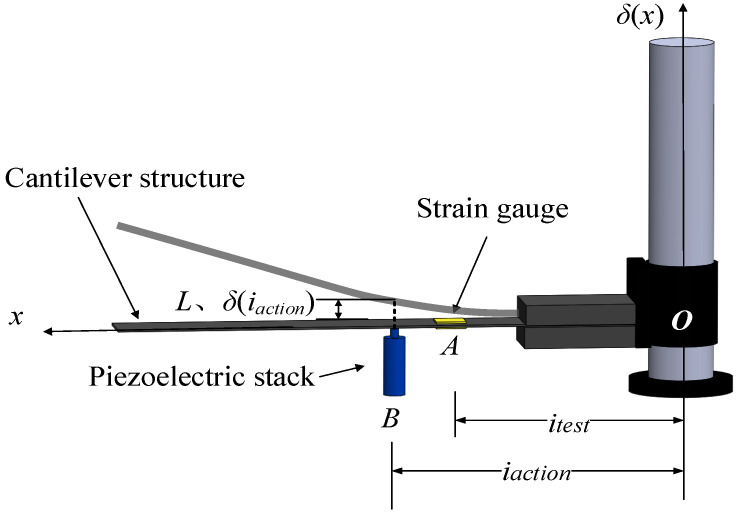
Experimental setup schematic for PSA load-bearing capacity characterization.

To evaluate the load-bearing capacity of PSAs, a custom cantilever structure was fabricated and integrated with the actuator [25]. The PSA was mounted beneath the beam near its fixed end, with a hemispherical tip contacting the beam surface. Electrical excitation signals were synthesized by a signal generator, subsequently amplified through a high-voltage amplifier (0–120 V), and applied to the PSA to induce controlled bending in the cantilever structure. Strain gauges bonded to the beam surface captured bending strain, which was processed through a digital strain acquisition system (Figure 1). The measured bending strain directly correlated with PSA load-bearing capacity, as higher excitation voltages amplified actuator output forces, thereby increasing strain magnitudes. This strain-voltage dependency validated the cantilever as a reliable metric for quantifying PSA performance.

The spatial configuration of the PSA actuation point (Point *B*) and strain sensor location (Point *A*) on the cantilever is adjustable to optimize measurement efficacy. Given the limited bending strain caused by micrometer-scale PSA displacements, acquired signals are susceptible to ambient noise interference. To amplify strain magnitudes and enhance signal fidelity, a strain transfer model was formulated based on Euler-Bernoulli beam mechanics. This model enables systematic optimization of PSA and sensor positioning to concurrently maximize strain amplitude and signal-to-noise ratio (SNR), ensuring robust data acquisition under coupled T-E-M fields.

As illustrated in Figure 1, the PSA-driven cantilever system is abstracted as a clamped-free beam with the fixed boundary denoted as Point *O*. Critical geometric parameters include:

1. Actuation interface distance *i_action_*: Separation between the PSA actuation point (Point *B*) and the anchored end (Point *O*).
2. Measurement node distance *i_test_*: Span from Point *O* to the strain sensor location (Point *A*).

This parametric framework facilitates precise correlation between PSA output characteristics and structural deformation metrics, essential for high-fidelity performance evaluation under multi-physical loading conditions.

Under predefined electrical excitation parameters and mechanical boundary constraints of the cantilever system, the PSA exhibits a displacement magnitude *L*. This electromechanical interaction generates a reaction force *F* at the clamped support while inducing a tip deflection *δ*(*i_action_*) at the PSA-cantilever interface (Point *B*). To analytically resolve the structural response, the Euler-Bernoulli beam theory was implemented, establishing a governing equation set that quantifies:

1. Deflection profile *δ*(*x*): Spatial variation of beam deflection along the longitudinal axis (coordinate *x*).
2. Flexural moment *M_A_*: Bending moment magnitude at the strain sensor location (Point *A*).

The aforementioned parameters are rigorously defined through the fundamental governing equations of beam flexural mechanics, formulated as follows:

(1) $$\delta (x)=\frac{Fx^{2}}{6EI}(3i_{action}-x),(0\leq x\leq i_{action})$$

(2) $$M_{A}=F(i_{action}-i_{test})$$

In the analytical framework, *E* denotes the elastic modulus of the cantilever material, while the second moment of inertia *I* is defined as *I* = *b·d* ^3^/12, where *b* and *d* represent the beam’s width and thickness, respectively. The constitutive relation between stress *σ* and strain *ε* follows *ε = σ*/*E*, with normal stress at the measurement node expressed as *σ* = *M_A_*/*W*. Here, *W* = (*b·d* ^2^)/6 corresponds to the bending section modulus. By substituting these relations into Equation (2), the strain *ε_A_* induced at Point *A* due to the PSA-generated force *F* is derived as:

(3) $$\varepsilon_{A}=\frac{F(i_{action}-i_{test})}{WE}$$

According to Equation (1), the deflection generated by the PSA at its actuation point is obtained as follows:

(4) $$\delta (i_{action})=L=\frac{F\cdot {\left(i_{action}\right)}^{3}}{3EI}$$

Following the electromechanical behavior of the PSA, the output force *F* corresponding to a displacement magnitude *L* is defined by the constitutive relationship:

(5) $$F=k(L_{o}-L)$$

Here, *L_o_* denotes the free-state displacement output of the PSA, and *k* represents its intrinsic stiffness. Through the synthesis of Equations (4) and (5), the resultant output force *F* is analytically derived as:

(6) $$F=\frac{3k\cdot IEL_{o}}{k\cdot {\left(i_{action}\right)}^{3}+3EI}$$

Consequently, the strain magnitude *ε_A_* at strain sensor location (Point *A*) is derived by substituting Equation (6) into Equation (3), resulting in:

(7) $$\varepsilon_{A}=\frac{3kIL_{o}(i_{action}-i_{test})}{W[k\cdot {\left(i_{action}\right)}^{3}+3EI]}$$

Equation (7) identifies *i_test_* and *i_action_* as primary variables governing strain response. Geometric parameters such as the beam’s moment of inertia *I* and bending section modulus *W* exhibit negligible variation within the operational thermal regime. Crucially, both the elastic modulus *E* of the cantilever and the PSA stiffness *k* demonstrate a thermally coupled enhancement with decreasing temperatures. This compensatory behavior effectively mitigates temperature-induced fluctuations in strain *ε_A_*, as validated by the reformulated expression of Equation (7):

(8) $$\varepsilon_{A}=\frac{3IL_{o}(i_{action}-i_{test})}{W[{\left(i_{action}\right)}^{3}+3I\frac{E}{k}]}$$

The theoretical model further reveals that a temperature reduction leads to a decline in the PSA’s free-state displacement output *L_o_*, as expressed in Equation (8). Consequently, the strain magnitudes *ε_A_* at the cantilever’s strain sensor location-which directly correlate with the PSA’s load-bearing capacity-diminish proportionally due to reduced actuation displacement. This displacement dependency also elucidates strain variations induced by prestress adjustments and excitation voltage modulation. Critically, the observed bending strain alterations are exclusively attributed to the PSA’s load-bearing capacity, confirming its dominance over other potential influencing factors.

For experimental validation, a PSA (model: PSt 150/10/40 VS15, Harbin Core Tomorrow Science and Technology Co., Ltd., Harbin, China) was integrated into the test setup. Key parameters of the PSA and cantilever assembly, including geometric dimensions and material properties, are tabulated in Table 1 based on manufacturer specifications.

### 2.2. Optimal Placement of Strain Sensor and Actuation Positions via Particle Swarm Optimization

#### 2.2.1. Formulation of Optimization Objectives and Constraints

A multi-objective optimization framework was developed to determine the optimal spatial coordinates of the strain sensor *i_test_* and PSA actuation interface *i_action_* on the cantilever beam, leveraging the theoretical strain model (Equation (7)) and the Particle Swarm Optimization (PSO) algorithm. The framework simultaneously targets maximization of the signal-to-noise ratio (SNR)-quantified as the logarithmic ratio of theoretical strain magnitude *ε_A_* to noise amplitude *ε_noise_* and enhancement of strain sensitivity. The SNR quantifies the robustness of the measured strain signal against environmental noise. It is derived from the theoretical strain value *ε_A_* and the noise amplitude *ε_noise_*:

(9) $$SNR(i_{test},i_{action})=20\log_{10}[\frac{\varepsilon_{A}(i_{test},i_{action})}{\varepsilon_{noise}}]$$

where *ε_noise_* = 2 *με* represents the fixed noise level in the experimental setup.

Sensitivity, reflecting the strain variation per unit output force, is derived from the partial derivative of *ε_A_* with respect to the piezoelectric stack output force *F*, yielding:

(10) $$S(i_{test},i_{action})=\frac{\partial \varepsilon_{A}}{\partial F}=\frac{i_{action}-i_{test}}{WE}$$

To balance these objectives, a weighted sum approach combines SNR and sensitivity into a unified objective function:

(11) $$f(i_{test},i_{action})=\alpha SNR(i_{test},i_{action})+\beta S(i_{test},i_{action})$$

The weighting coefficients (*α* = 0.7, *β* = 0.3) prioritize SNR to mitigate low-temperature noise interference, while retaining sufficient sensitivity for dynamic load resolution in practical applications. This allocation aligns with optimization heuristics for high-noise multiphysics systems, ensuring robust signal fidelity under extreme coupled-field conditions.

The optimization framework incorporates two critical constraints to ensure system integrity: (1) Geometric feasibility mandates a minimum separation distance of 10 mm between the PSA actuation interface *i_action_* and strain sensor location *i_test_* to prevent spatial interference; (2) Mechanical safety limits the maximum permissible strain at the sensor location to 500 με, mitigating the risk of material yielding under operational loads. These constraints are algorithmically enforced throughout the PSO iterations to guarantee physically realizable and failure-resistant configurations.

This methodology seamlessly integrates theoretical predictions with empirical feasibility, offering a systematic approach to improve measurement precision and operational reliability of PSA-driven systems operating under coupled T-E-M environments.

#### 2.2.2. Implementation of Particle Swarm Optimization

The PSO algorithm is employed to solve the constrained optimization problem, with the following steps:

(1) Particle Definition: Each particle in the swarm represents a candidate solution *x*_*i*_ = (*i*_*test*_, *i*_*action*_), where *i*_*test*_ ∈ [20 mm, 50 mm] and *i*_*action*_ ∈ [30 mm, 60 mm]. The velocity vector *v*_*i*_ = (*v*_*itest*_, *v*_*iaction*_) governs the search direction. A population of *N* = 30 particles is randomly initialized within the feasible domain.
(2) Fitness Evaluation: The fitness of each particle is quantified by evaluating the objective function *f* (*i_test_*, *i_action_*), which integrates SNR and sensitivity metrics. Solutions violating constraints are subjected to penalty mechanisms: particles exceeding strain limits are reinitialized within feasible regions, while those violating spatial separation are dynamically repositioned.
(3) Iterative Position-Velocity Update: The swarm’s exploration-exploitation balance is maintained through velocity and position updates at each iteration:

(12) $$x_{i}^{t+1}=x_{i}^{t}+v_{i}^{t+1}$$

(13) $$v_{i}^{t+1}=wv_{i}^{t}+c_{1}r_{1}(P_{best,i}-x_{i}^{t})+c_{2}r_{2}(g_{best}-x_{i}^{t})$$

The inertia weight *w* = 0.7 balances global exploration and local exploitation, while acceleration coefficients *c*_1_ = *c*_2_ = 1.5 scale the attraction forces toward the particle’s historical best position *P_best_* and the swarm’s global best *g_best_*. Stochasticity is introduced via uniformly distributed random numbers *r*_1_, *r*_2_ ~ *U* (0, 1), ensuring search diversity.

(4) Constraint Handling Mechanism: If the PSA actuation interface violates the minimum spatial separation requirement *i_action_* < *i_test_* + 10 mm, the particle is dynamically repositioned to *i_action_* = *i_test_* + 10 mm + *δ*, where *δ* ~ *U* (1 mm, 5 mm) introduces stochastic displacement compensation; Particles exceeding the strain limit (*ε_A_* ≥ 500 *με*) are immediately discarded and regenerated within the feasible domain to prevent non-physical solutions.
(5) Termination Criteria: The algorithm terminates after *T* = 100 iterations or when the objective function converges (relative change < 0.1% over 10 iterations).

**Figure 2:**
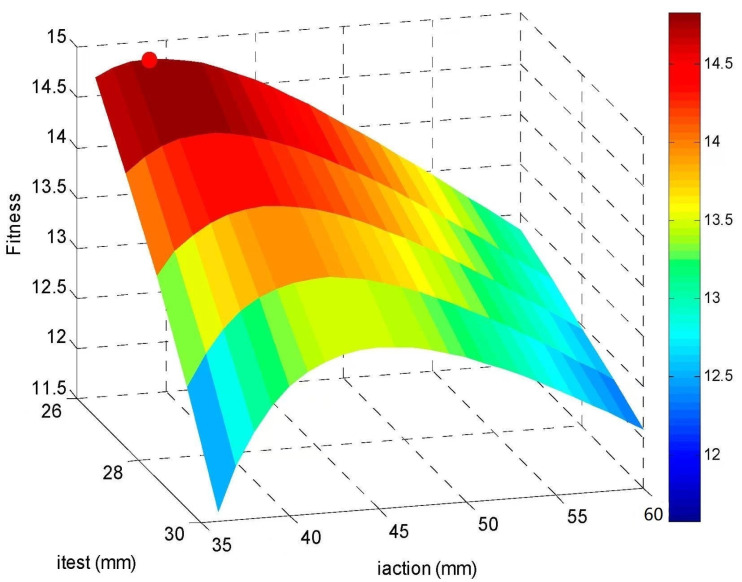
Three-dimensional distribution of the objective function varying with the test length (*i_test_*) and action length (*i_action_*).

The PSO algorithm demonstrates efficient navigation of the design space, maintaining an equilibrium between global search breadth and local refinement precision. Through the weighted multi-objective function, a Pareto-optimal configuration is identified, achieving maximal signal-to-noise ratio while preserving strain sensitivity. The results show that the optimized positions *i_test_* = 26 mm and *i_action_* = 39 mm achieve a strain signal amplitude of 14.7 με with an SNR of 14.8 dB. The three-dimensional objective function landscape (shown in Figure 2), plotted as a function of sensor (*i_test_*) and actuation (*i_action_*) positions, further confirms that the fitness achieves peak (marked by the red dot in Figure 2) when positioned 39 mm from the clamped boundary. This configuration simultaneously maximizes strain magnitude and SNR.

## 3. Experimental Characterization of PSA Load-Bearing Capacity Under Multi-Physical Coupling

### 3.1. Low-Temperature Mechanical Instrumentation Design

**Figure 3:**
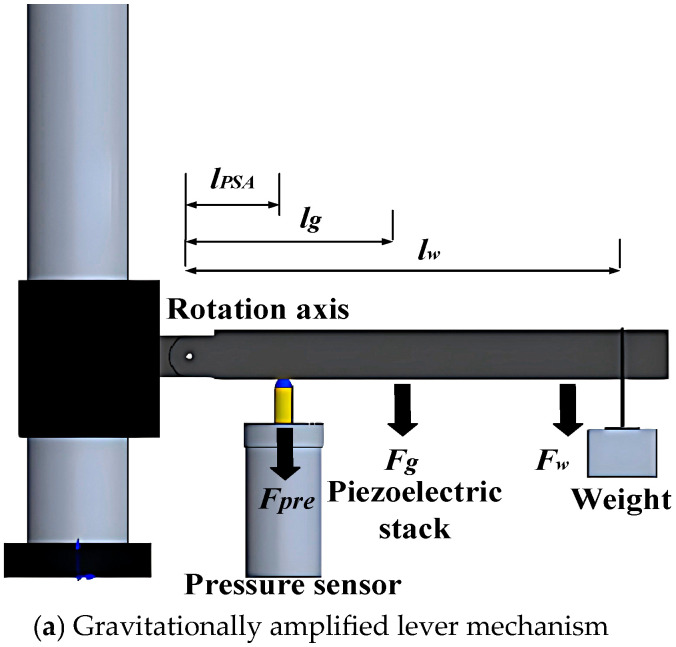

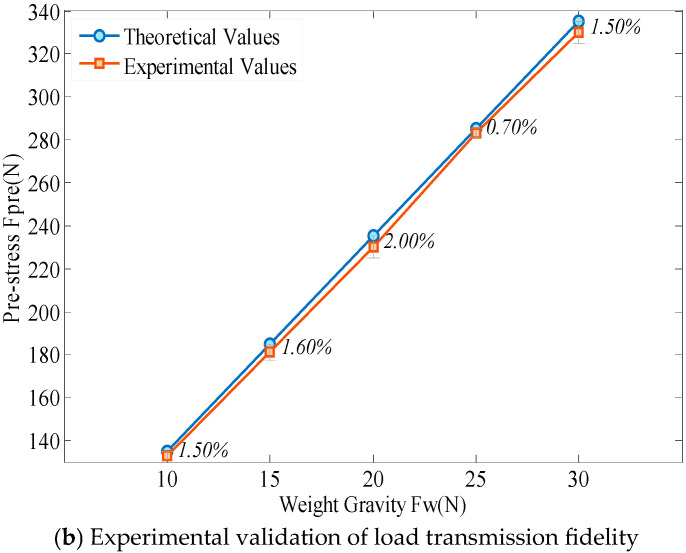
Lever-based prestress application mechanism and experimental validation.

To quantify the interdependent effects of low-temperatures (down to −70 °C), compressive prestress (0 to 6 MPa), and electrical excitation (0 to 120 V) on the PSA’s load-bearing capacity, a custom instrumentation system was engineered with two core capabilities: 1. Temperature-invariant prestress application: A lever-based loading mechanism (Figure 3a) generates stable mechanical preloads via gravitational force amplification, immune to thermal variations. 2. Low-temperature performance evaluation: Characterization of the PSA’s load-bearing capacity could be conducted at sub-zero temperatures.

The lever system comprises a steel arm and calibrated weights, designed to amplify applied masses through moment equilibrium. Under static mechanical equilibrium, the prestress *F_pre_* is derived from the torque balance equation:

(14) $$F_{pre}=\frac{F_{g}l_{g}+F_{w}l_{w}}{l_{PSA}}$$

where: *F_w_*, *F_g_*—Gravitational forces of the weight and lever, respectively. *l_w_*, *l_g_*, *l_PSA_*—Moment arms from the rotation axis to the weight, lever’s center of mass, and PSA interface. Prestress magnitude is adjustable by varying the weight mass *F_w_* or adjusting the lever arm ratio *l_w_*/*l_PSA_*. The rotational joint (stiffness: 2.1 × 10^9^ N/m) ensures minimal bending deformation satisfying the rigid-body assumption in torque calculations. Crucially, the gravitational preload remains unaffected by thermal contraction, enabling decoupled analysis of temperature and mechanical effects.

Equation (14) is formulated under the rigid-body assumption, where the lever undergoes negligible bending deformation. To validate this assumption, the lever’s geometric and material properties including its square cross-section (16 × 16 mm^2^), Steel 45 composition, and axial stiffness (1.5 × 10^8^ N/m) were designed to minimize flexural strain. Experimental parameters were configured as follows: Lever arm lengths: *l_PSA_* = 35 mm, *l_w_* = 350 mm, *l_g_* = 175 mm; Gravitational forces: Lever self-weight *F_g_* = 7 N; applied weights *F_w_*= 10, 15, 20, 25, 30 N. Theoretical prestress values, calculated via Equation (14), were compared against experimental measurements obtained from a high-stiffness pressure sensor (1 × 10^7^ N/m). As summarized in Figure 3b, the close agreement between theoretical and experimental results (deviation < 2%) confirms the validity of the rigid-body assumption and demonstrates precise prestress control through weight adjustment, with bending strains remaining below detectable thresholds (<1 *με*).

**Figure 4:**
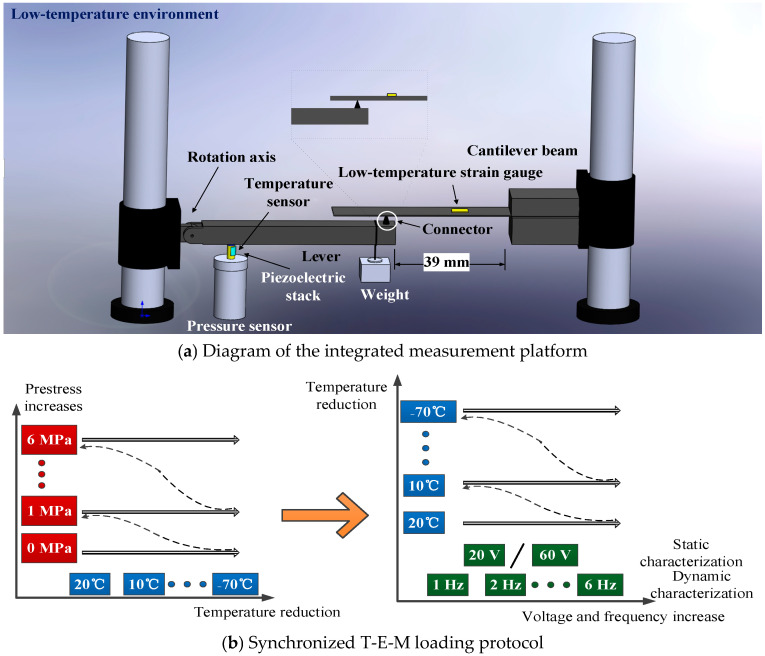
Integrated multiphysics characterization platform.

Given the negligible bending strain on the lever and the impracticality of deploying laser displacement sensors in low-temperature environments, the PSA’s load-bearing capacity under low-temperature conditions was indirectly characterized through a coupled lever-cantilever system. As illustrated in Figure 4a, the PSA-driven lever tip interfaces with the free end of the optimized cantilever (sensor position: 26 mm, actuation position: 39 mm) via a low-friction connector. This connector maintains only minimal contact with the cantilever’s free end, strictly ensuring that gravitational loads from the lever do not influence beam deformation.

When the PSA is electrically excited under prestress, its vertical displacement elevates the lever tip, inducing upward bending in the cantilever. The resulting strain field, monitored by low-temperature strain gauges bonded to the cantilever surface, serves as a direct metric of the PSA’s load-bearing capacity. This methodology enables constant prestress application in low-temperature environments while maintaining measurement fidelity through indirect strain assessment.

The experimental protocol was executed in sequential phases to systematically evaluate PSA performance under coupled T-E-M conditions: 1. Prestress and Temperature Cycling: Initial tests commenced with zero prestress, followed by incremental increases (100 N steps, 0–600 N range, equivalent to 0–6 MPa) after each temperature cycle, prestress levels remained below 10% of the PSA’s blocking force (7000 N) to prevent mechanical fatigue. Temperature was cycled from 20 °C to −70 °C in 10 °C decrements, with strain measurements recorded at each interval using static strain instrumentation. After reaching −70 °C, the system was reverted to ambient temperature for prestress adjustment, as shown in Figure 4b. 2. Electromechanical Excitation Modes: Static Characterization: DC voltages of 60 V and 120 V were applied to induce static deformations, with strain data acquired via static strain analyzers. Dynamic Characterization: Sinusoidal AC voltages (120 V amplitude, 1–6 Hz frequency sweep) excited the PSA, while dynamic strain recorders captured transient responses.

**Figure 5:**
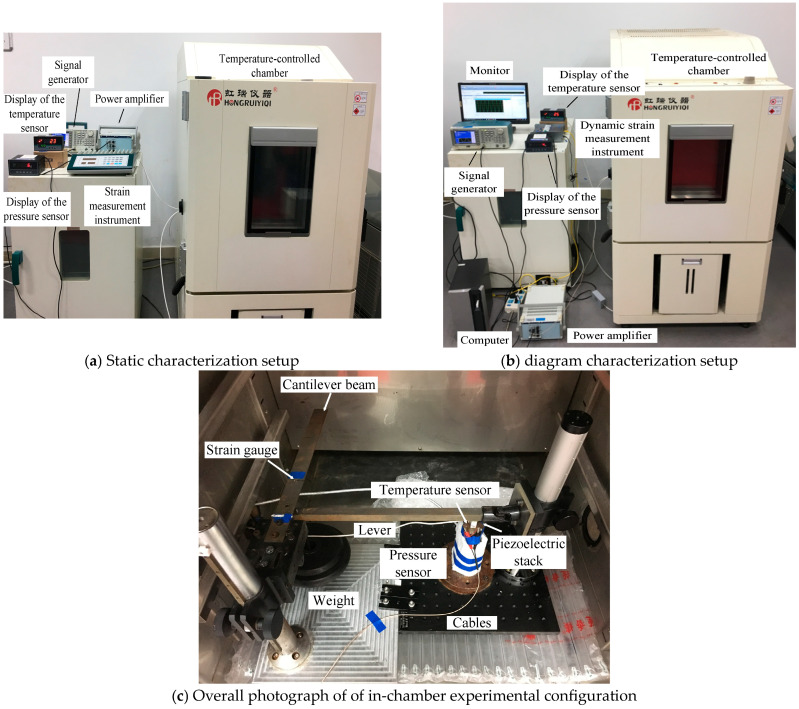
Multiphysics experimental characterization of the PSA’s load-bearing capacity under coupled thermal, mechanical, and electrical stimuli.

The experimental assembly was housed within a temperature-controlled chamber (dimensions: 500 × 600 × 700 mm^3^) capable of precise thermal regulation from −70 °C to +100 °C, with temperature stability maintained at ±0.2 °C (deviation) and ±0.5 °C (fluctuation). Sensor power supplies and data acquisition units were externally mounted, while instrumentation cabling connecting internal components to external systems was routed through sealed ports on the chamber wall. Electrical excitation was provided by a arbitrary waveform generator (model: AFG3021C, Tektronix, Inc., Beaverton, OR, USA), amplified through a high-voltage amplifier (model: XE501-A, Harbin Core Tomorrow Science and Technology Co., Ltd., Harbin, China, specifications in Table 2), while the temperature of the PAS was monitored via embedded PT1000 RTD sensors (model: PT1000, SenseCraft Technology Co., Ltd., Xi’an, China), and the complete experimental configuration is illustrated in Figure 5.

This integrated setup enabled synchronized control of mechanical preload, electrical excitation, and temperature during PSA characterization, ensuring precise correlation between input parameters and measured load-transfer dynamics.

### 3.2. Characterization of Coupled Thermo-Electro-Mechanical Response

#### 3.2.1. Static Load-Bearing Capacity Analysis

**Figure 6:**
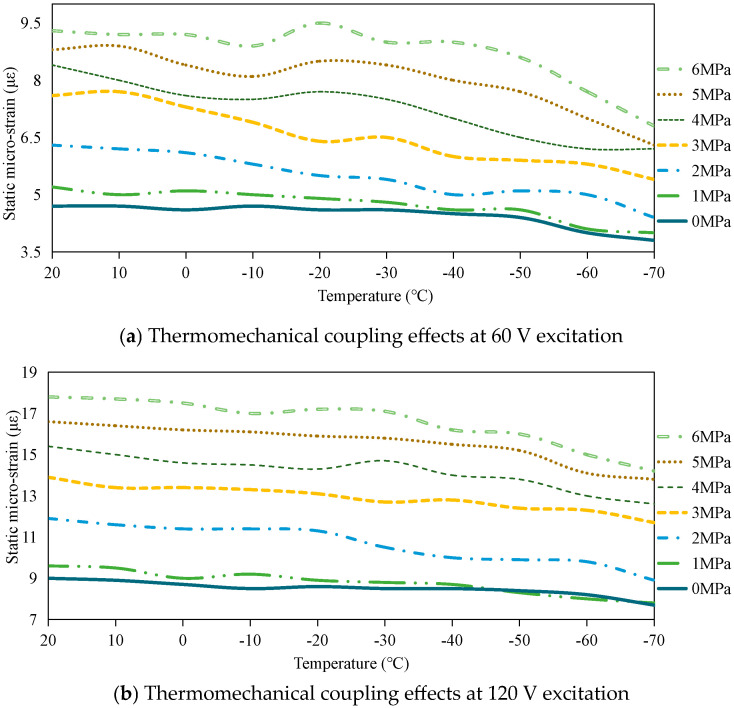
Static load-bearing capacity under thermomechanical coupling and varied excitation voltages.

Under varied excitation voltages of 60 V and 120 V, experimental investigations were conducted to evaluate the temperature- and prestress-dependent mechanical performance of a piezoelectric stack, as illustrated in Figure 6. The data demonstrate that under equivalent environmental and mechanical constraints, the strain magnitude at 120 V excitation consistently doubles that observed at 60 V. This proportional enhancement confirms a voltage-dependent linear augmentation in the load-bearing capacity, which remains unaffected by thermal variations or mechanical preload adjustments.

Experimental observations further reveal two distinct trends: First, elevated prestress levels enhance the load-bearing capacity under constant thermal conditions. Second, reduced ambient temperatures generally diminish this capacity under fixed prestress values, though minor anomalies were detected in isolated measurements. These deviations (confined within 5% variation) may be attributed to two factors: temperature-induced instability of piezoelectric coefficients within the −20 °C to 20 °C range, as documented in prior research [15], and inherent measurement uncertainties across experimental trials. Notably, such fluctuations do not compromise the overall data trends.

Quantitative analysis indicates a 15–30% reduction in static micro-strain when temperature decreases from 20 °C to −70 °C. Conversely, increasing prestress from 0 MPa to 6 MPa produces 79–90% micro-strain enhancement. These proportional relationships between electromechanical parameters (excitation voltage, temperature, prestress) and mechanical response validate the deterministic nature of the stack’s load-bearing capacity under specified operational conditions.

The observed correlations between load-bearing capacity and operational parameters (prestress, temperature) align with established relationships governing material properties [26,27] and electromechanical outputs [28] in preloaded PSA. These interdependencies originate from fundamental material responses, as substantiated by prior structural analyses [15,24,29]: Enhanced prestress promotes non−180° domain boundary mobility under high electric fields, thereby amplifying piezoelectric coefficients through intensified domain switching. This microstructural activation elevates both electromechanical outputs and load-bearing capacity, provided preload levels remain below depolarization thresholds.

Concurrently, thermal reduction diminishes piezoelectric responsiveness through constrained domain wall motion [22], explaining the temperature-dependent capacity reduction demonstrated in Figure 6. These phenomena collectively demonstrate how T-E-M coupling modifies critical material characteristics: alterations in domain configurations and crystalline phase states at microstructural levels directly govern macroscopic stack performance. Such structural modifications propagate through the system hierarchy, ultimately dictating functional outputs (displacement generation, force transmission) that determine load-bearing capacity. These agreements-spanning fundamental piezoelectric principles and prior literature-collectively validate the methodology through rigorous cross-verification.

**Figure 7:**
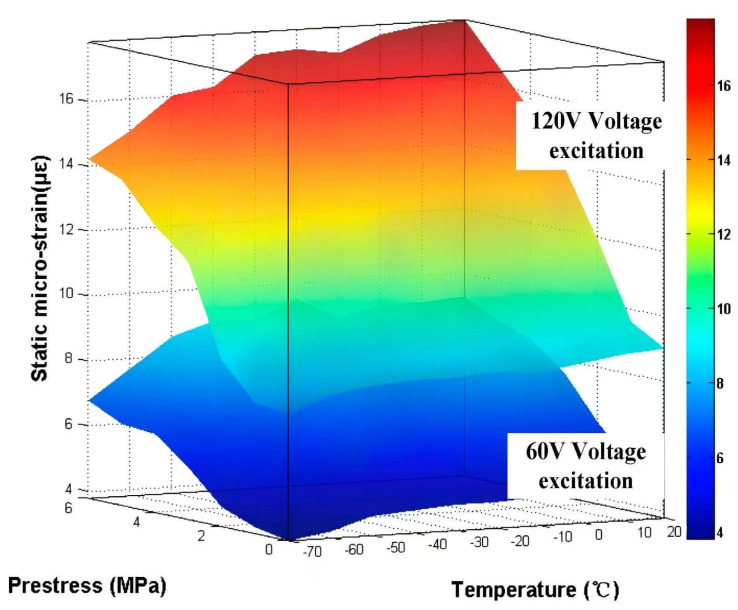
Comprehensive 3D response surface characterizing PSA load-bearing capacity under T-M-E excitation.

**Figure 8:**
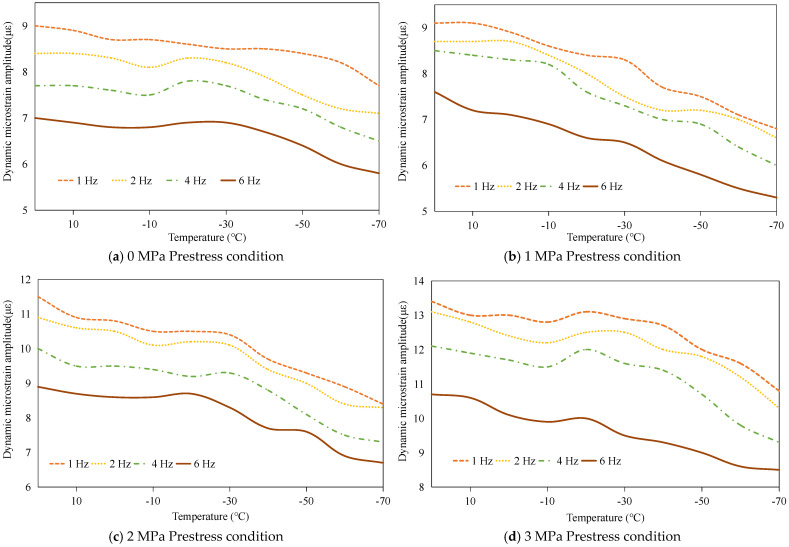

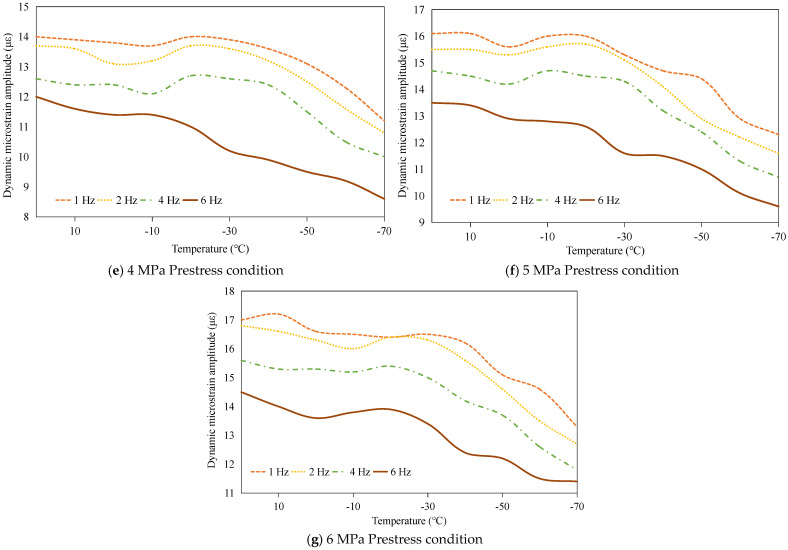
Frequency- and temperature-dependent dynamic load-bearing capacity of the PSA under varying prestress conditions.

Figure 7 comprehensively illustrates these multidimensional interactions through a three-dimensional response surface mapping capacity variations against combined thermal, mechanical, and electrical parameters. Figure 8 illustrates the three-dimensional parametric response surface characterizing the static load-bearing behavior of the piezoelectric stack as functions of temperature (−70 to 20 °C), prestress (0–6 MPa), and excitation voltage (60 and 120 V).

#### 3.2.2. Dynamic Load-Bearing Capacity Analysis

**Figure 9:**
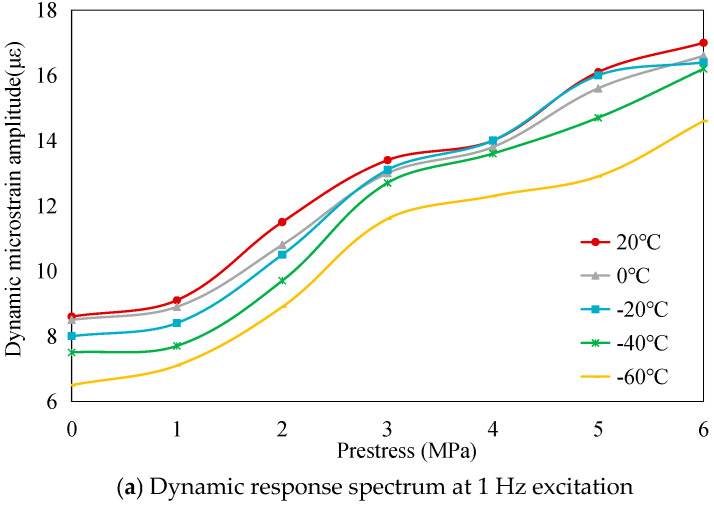

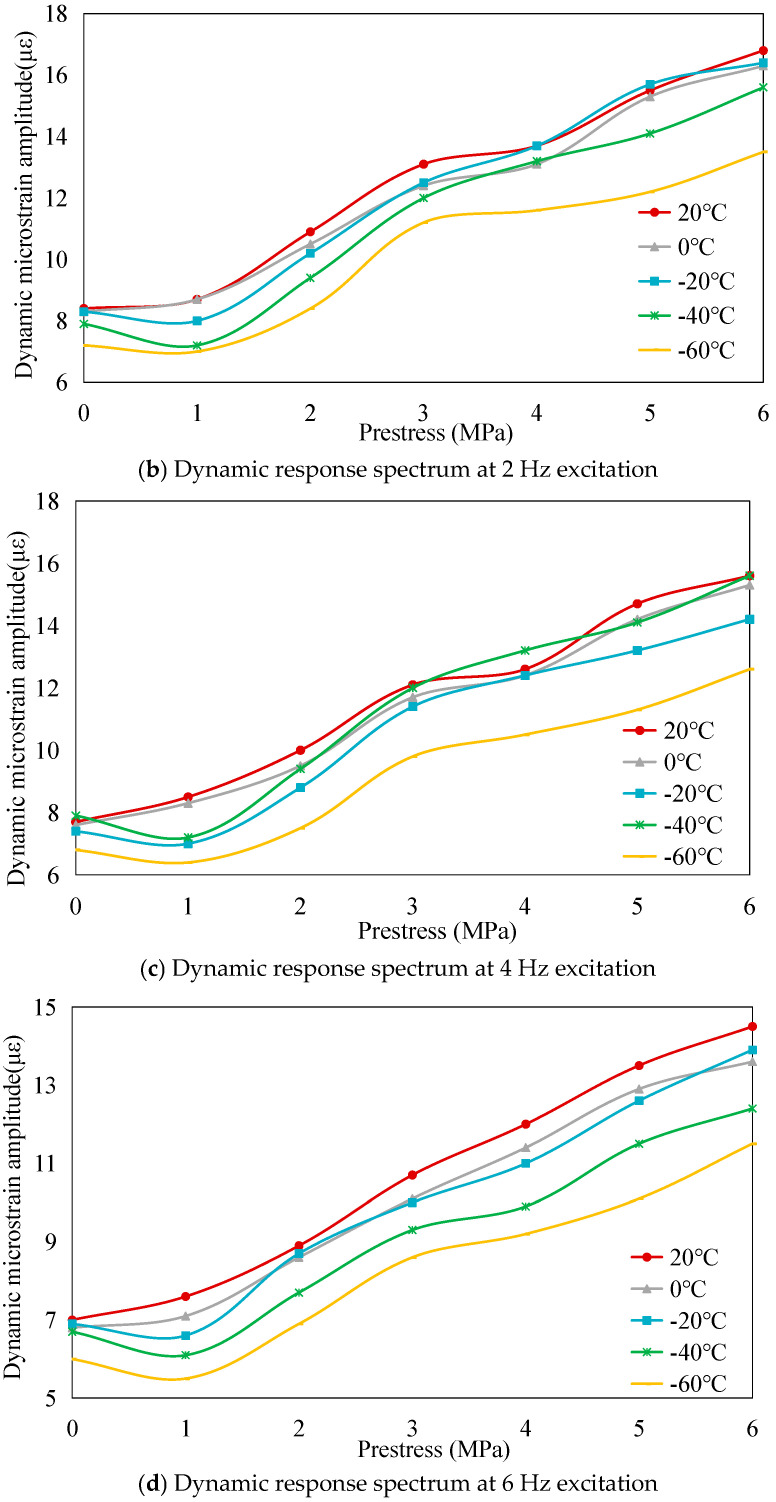
Frequency-dependent dynamic load-bearing capacity of the piezoelectric stack under coupled thermomechanical conditions (6 Hz excitation).

The dynamic load-bearing capacity of the PSA was systematically investigated through cyclic voltage excitation tests. Sinusoidal AC signals (120 V amplitude, 1–6 Hz frequency range) were applied to quantify temperature- and prestress-dependent variations in load-bearing capacity, with dynamic responses characterized by micro-strain peak-to-peak magnitudes. Experimental data reveal three principal trends: First, frequency-dependent attenuation occurs across the tested spectrum, as depicted in Figure 8. This behavior stems from dual constraints-peak current limitations in power amplification systems and finite response velocities inherent to piezoelectric materials. Second, parametric investigations demonstrate prestress-enhanced capacity under isothermal conditions and thermal-diminished performance under fixed mechanical preloads (Figure 9), mirroring static load-bearing trends established in prior analyses. These congruent patterns across static and dynamic regimes confirm the robustness of prestress- and temperature-dependent relationships in governing stack performance.

Critically, these T-E-M-coupled phenomena-which commercial instrumentation (e.g., Aixacct AIXCMA, aixACCT mechatronics Co., Ltd., Aachen, Germany) cannot capture due to limitations in prestress integration and load-bearing capacity quantification-demonstrate our methodology’s ability to characterize voltage-dependent load-bearing capacity under multiphysics conditions, complementing standardized parameter measurements.

## 4. Conclusions

This study establishes the first methodology for quantifying the load-bearing capacity of PSAs under multiphysics-coupled conditions at low-temperature through the development of novel instrumentation. The instrumentation architecture incorporates a mechanically optimized cantilever system with analytical positioning of strain sensors and actuation interfaces, validated via particle swarm optimization to maximize strain signal fidelity. Experimental characterization under synchronized thermal (−70 °C to 20 °C), mechanical (0–6 MPa prestress), and electrical (DC/AC, 0–120 V, 1–6 Hz) controls revealed three critical interdependencies: (1) A voltage-dependent linear enhancement of load-bearing capacity across all tested conditions, demonstrating excitation dominance over environmental factors; (2) Prestress-induced amplification (79–90% increase from 0 to 6 MPa) and thermal degradation (15–30% reduction from 20 °C to −70 °C) of static load-bearing capacity; (3) Dynamic performance consistency with static trends, albeit exhibiting frequency-dependent attenuation (1–6 Hz). These results provide the first experimental quantification of multiphysics-coupled load-bearing capacity, resolving the critical knowledge gap. The proposed methodological framework establishes foundational principles for developing integrated measurement systems capable of quantifying T-M-E coupling effects on piezoelectric stack performance across operational domains spanning thermal gradients, mechanical prestress, and electrical excitation parameters. While validated for PSAs, the methodology extends to longitudinal-mode piezoelectrics via parameter recalibration, with broader applicability achievable through fixture adaptations guided by the Euler-Bernoulli framework. The methodology’s scalability to broader parameter ranges and material systems positions it as a benchmark for future investigations of smart material behavior under extreme multiphysics conditions.

## Figures and Tables

**Table 1:** Geometric and material parameters of cantilever-piezoelectric assembly.

Description	Parameter	Value	Unit
Stiffness of the PSA	*k*	6 × 10^7^	N/m
Output displacement of the PSA under free state	*L_o_*	3.5 × 10^−5^	m
Electrical excitation range	*U_PSA_*	0~120	V
Length of the PSA	*L_PSA_*	4.6 × 10^−2^	m
Width of the cantilever	*b*	3 × 10^−2^	m
Thickness of the cantilever	*d*	2 × 10^−3^	m
Elastic modulus of the cantilever	*E*	2.1 × 10^11^	Pa

**Table 2:** Parameters of the power amplifier.

Description	Value	Unit
Average current	60	mA
Peak current	180	mA
Average power	7	W
Bandwidth	1	kHz
Voltage gain	12	-
Input voltage range	0~10	V
Output voltage range	0~120	V

## Data Availability

Restrictions apply to the availability of these data. The datasets presented in this article are not readily available because of company restrictions. Requests to access the datasets should be directed to the corresponding author.
